# DNA Methylation Program in Developing Hippocampus and Its Alteration by Alcohol

**DOI:** 10.1371/journal.pone.0060503

**Published:** 2013-03-27

**Authors:** Yuanyuan Chen, Nail Can Ozturk, Feng C. Zhou

**Affiliations:** 1 Department of Anatomy and Cell Biology, Indiana University School of Medicine, Indianapolis, Indiana, United States of America; 2 Stark Neuroscience Research Institute, Indiana University School of Medicine, Indianapolis, Indiana, United States of America; 3 Department of Anatomy, Mersin University, Mersin, Turkey; Universitat Pompeu Fabra, Spain

## Abstract

During hippocampal development, the Cornus Ammonis (CA) and the dentate gyrus (DG) undergo waves of neurogenesis and neuronal migration and maturation independently. This stage is widely known to be vulnerable to environmental stresses, but its underlying mechanism is unclear. Alcohol exposure has been shown to alter the expression of genes that regulate the fate, survival, migration and differentiation of pyramidal and granule cells. Undermining this process might compromise hippocampal development underlying the learning and memory deficits known in Fetal Alcohol Spectrum Disorders (FASD). We have previously demonstrated that DNA methylation was programmed along with neural tube development. Here, we demonstrated that DNA methylation program (DMP) proceeded along with hippocampal neuronal differentiation and maturation, and how this DMP was affected by fetal alcohol exposure. C57BL/6 mice were treated with 4% v/v ethanol through a liquid diet along with pair-fed and chow-fed controls from gestation day (E) 7 to E16. We found that a characteristic DMP, including 5-methylcytidine (5mC), 5-hydroxylmethylcytidine (5hmC) and their binding proteins, led the hippocampal neuronal differentiation and maturation spatiotemporally as indicated by their phenotypic marks in the CA and DG pre- and post-natally. Alcohol hindered the acquisition and progression of methylation marks, and altered the chromatin translocation of these marks in the nucleus, which was correlated with developmental retardation.

## Introduction

Maternal alcohol intake during pregnancy adversely affects the developing fetus, leading to various degrees of developmental deficits and growth retardation, collectively referred to as Fetal Alcohol Spectrum Disorders (FASD). One of the signature, as well as the most severe, consequences is the brain deficit and accompanying cognitive and neurobehavioral disorder, which often persist into adulthood. The hippocampus is a key brain region of these functions and is one of the most vulnerable regions to ethanol-induced neurotoxicity. Children with FASD often have memory impairment, learning deficits, and affective disorders [1]. Rats experimentally exposed to a high dose of ethanol during early postnatal life showed a long-lasting deficit in spatial learning ability and memory formation [2]. Collaborative reports have shown that fetal alcohol exposure leads to reduced dentate gyrus volume, granule cell number, dendritic arborization and neurite outgrowth of granule cells and pyramidal cells; and decreased levels of neurotrophic factors BDNF and NGF, as well as stem cell proliferation in DG [3], [4], [5], [6], [7]. However, the mechanism underlying alcohol-induced abnormality of hippocampal formation still remains unknown. Recently, we have found that alcohol actively alters epigenetic programming during neural tube development, and inhibiting epigenetics at the same stage mimics the alcohol induced growth retardation in many organs including the brain, the heart, and the cranioface [8].

Epigenetics is codes written on (but not in) DNA and histones in the chromatin, which regulate transcription by modifying 3D conformation and accessibility (to transcription related binding proteins) of DNA. DNA methylation (5mC) is written by DNA methylation transferase (DNMT) on cytosine [9]. The formation of 5mC is associated with condensation of DNA and suppression of gene transcription [10]. The methylation on cytosine is not fixed, nor random; it is emerging as a critical mediator for development. It has been found that DNA Methylation appears as an orderly Program (DMP) which precedes and mediates development from embryonic stem cells[11] to the neural tube[8] (for review see [12]). Whether there is a DMP directing brain development, such as hippocampus development beyond the neural tube development, has not been known.

Passive demethylation occurs during cell replication where lack of maintenance to DNMTs leads to depletion of methylation at the newly synthesized DNA strand. Active demethylation is also found and dynamically regulated in post-mitotic cells, e.g. CA1 pyramidal neurons and DG granule cells, and has been shown to modulate synaptic plasticity and memory consolidation [13], [14], [15]. In active demethylation, the 5mC is converted to 5hmC by ten-eleven translocation 1/2/3 (TET1/2/3) enzymes [16]. However, instead of as a demethylation intermediate, the 5hmC is gaining evidence to play an important role in maintaining pluripotency in embryonic stem cells along with neuronal maturation (for review see[17] ). The 5hmC is more abundant in the nervous system than in any other tissues [18], and its presence in the gene body is associated with gene expression in differentiated neural tissues in vitro [19], [20]. In mouse cortex and cerebellum, enrichment of 5hmC is associated with increased gene transcription level [20], [21] and specifically activation of synapse-relevant genes [22]. It has been shown that 5hmC prevents binding of DNMT1 to targeted cytosine, and subsequently facilitates gene transcription by suppressing binding of transcriptional repressors in embryonic stem cells [23], [24]. Despite these recent findings, the function of 5hmC in the brain is still unknown. Here we demonstrated, for the first time, that the transition of 5mC to 5hmC in the developing brain is correlated with neuronal differentiation and maturation.

Epigenetics also connects the genome to environmental signals and dynamically responds to environmental input without changing the genetic code (for review see [12]). Aberrant DNA methylation often leads to cancer by silencing of tumor suppressor genes [25]. Mutations in methyl-binding proteins also lead to the onset of many neurodevelopmental diseases, e.g. Rett syndrome [26], [27], [28]. How alcohol affects DMP in the hippocampus and impacts its development is a theme of this study. Alcohol, known as a methyl donor inhibitor, has been shown to inhibit the metabolic pathway of several methyl donors. In animals with fetal alcohol exposure, alcohol induced alterations in folate and homocysteine homeostasis, reduced methyl donor levels as well as DNMT proteins and mRNA levels in the brain. We have shown previously bi-directional, genome-wide methylation alterations in early prenatal alcohol treated mice embryos [29], correlated with altered gene expression [30]. Alcohol also disrupted the dorsal-ventral methylation gradation along neural tube maturation [8]. Here we investigate how prenatal alcohol exposure affects DNA methylation program in early hippocampal development.

## Materials and Methods

### Animals

All mice were used in accordance with National Institute of Health and Indiana University Animal Care and Use (IACUC) guidelines. The protocol was approved by the *Laboratory Animal Resource Center* (*LARC*) animal ethics committee of Indiana University (protocol ID: 10428). All efforts were made to assure minimal pain and discomfort. C57BL/6 (B6) (12–14 weeks old, 20 g ±1) nulliparous female mice (Harlan, Inc., Indianapolis, IN) were used in the study. Mouse breeders were individually housed upon arrival and acclimated for at least one week before mating began. The mice were maintained on a 12 hrs light-dark cycle (light on: 22:00–10:00) and provided laboratory chow and water ad libitum.

### Treatment Groups and Liquid Diet Administration

Mice were randomly assigned into three treatment groups: Chow, Pair-Fed (PF) and Alcohol (Alc). All alcohol treatments received 4% alcohol v/v in liquid diet [Purina Micro-Stabilized Alcohol Diet (PMI), Purina Mills Inc., Richmond, Indiana] as instructed by supplier with 4.4% w/v sucrose added, and administered using a 35 ml drinking tube (Dyets Inc, NY). The PF group was given the PMI diet mixture with equal caloric maltose dextran (MD) (Isocaloric diets) as a substitute for alcohol calories, and the volume of liquid diet intake was restricted to that of a matched dam from the alcohol group throughout all treatments. The Chow group maintained a standard chow diet and water ad libitum throughout gestation. The PF and Alc groups were pre-treated with liquid diet for 7 days before mating. Then females were bred with male breeders for a 2-hr period (10:00am to 12:00 noon). The presence of a vaginal plug at the end of the 2-hr mating was considered as indicative of conceptus and that hour was designated as hour 0, and embryonic day (E) 0. All animals were mated daily over a period of no more than 3 weeks, at which time all animals were on ad libitum chow and water diets, until plugs were detected. On E5, pregnant dams in PF and Alc groups were placed on liquid diet, and started to receive 4% v/v alcohol (Alc group) or isocaloric liquid diet (PF group) as indicated above from E7 through the end of E16. On E17, dams were either euthanized for embryo harvesting or were returned to standard lab chow and water and allowed to give birth. Additional E16 embryos from Chow group were also harvested for stage comparison. The postnatal pups were all nursed at birth by surrogated Chow-fed dams until the days of tissue harvesting. E19 was designated as postnatal day (P) 0 regardless if birth took place. Following birth, litters were randomly culled to six pups/litter to decrease possible nutritional deficiencies due to within-litter competition. Pups were then weighed daily and observed for any gross malformations until sacrifice on P7 (E26). The E17 and P7 stage were used for the study for their critical morphological progression in CA (E17 and P7) and in dentate gyrus (P7) development, and for their vulnerability to alcohol. Additional time point, P21, was also used to extend the observation after maturation.

### Blood Alcohol Concentrations

An independent set of non-pregnant females (n = 6) receiving 4% v/v alcohol drinking as in the above paradigm were used for blood alcohol concentration (BAC) analysis. Blood samples were collected through tail vein at 2 hrs or 6 hrs into the dark cycle on alternate days 2, 4 and 6 during treatment. 15 µl of blood was collected in heparinized tubes, and plasma was isolated through centrifugation and stored at −80°C prior to analysis with a Gas Chromatograph (GC, Agilent Technologies; model 6890). Each sample was analyzed in duplicate.

### Embryo Isolation and Tissue Preparation

Under deep CO_2_ euthanasia, embryos were harvested from dams at E17 by removal from the embryonic sack. Each embryo was either immersion-fixed in 20 ml of fixative prepared from 4% paraformaldyhyde (PFA) and stored at 4°C in the same fixative. For P7 group, pups were anaesthetized with CO_2_, and then perfused transcardially with 0.9% saline (100 ml) and 4% formaldehyde in phosphate buffer (0.2 M, pH∶7.4). The brains were then removed, weighed, and post-fixed for at least 24 hrs at 4°C.

### Immunocytochemistry Analysis

One Alc and either one PF or Chow brain were embedded in a single 10% gelatin block with careful rostrocaudal and dorsoventral alignments. Gelatin blocks were fixed with 4% PFA and sectioned in 40 µm thick coronal sections on a vibratome (Leica Microsystems; Buffalo Grove, IL). The section-pairs (Alc-PF or Alc-Chow) were processed equally in all immunocytochemical procedures. The section pairs were then cleared of endogenous peroxidases using a 10% H_2_O_2_ in phosphate buffered saline (PBS) for 10 min, and permeabilized with 1% TritonX-100 in PBS for 30 min before incubation with a primary antibody diluted in goat kit (1.5% goat serum, 0.1% TritonX-100 in PBS) for 18 hrs at room temperature. Antibodies used in this study were: 5mC (1∶2000, mouse monoclonal; Eurogenetec, Fremont, CA), 5hmC (1∶3000, rabbit monoclonal; Active Motif, Carlsbad, CA), TET1(ten-eleven translocation, a 5hmC hydroxylation enzyme; 1∶500, rabbit polyclonal; Millipore, Billerica, MA), MeCp2 (methyl CpG binding protein, a DNA methylation binding protein; 1∶1000, rabbit monoclonal; Cell Signaling, Danvers, MA), NeuN (a marker for mature neuron; 1∶500, cell signaling, Danvers, MA), Sox2 (a marker for neural progenitor cells; 1∶500, Millipore, Billerica, MA) and Ki67 (a marker for proliferating cells; 1∶1000, rabbit polyclonal; Abcam, Cambridge, MA). The section pairs were then incubated for 90 min in goat anti-rabbit IgG or goat anti-mouse secondary antibodies conjugated with biotin (Jackson ImmunoResearch, West Grove, PA) or fluorescent dyes (Invitrogen; Grand Island, NY), followed by Streptavidin-AP (1∶500, Jackson ImmunoResearch, West Grove, PA) for 90 min. The immunostaining was visualized by incubation of 0.05% 3′-3′-diaminobenzidine (DAB) and 0.003% H_2_O_2_ over minutes, followed by counterstained with methyl green. Fluorescent double staining was performed for colocalization studies (5hmC/ Sox2). Secondary antibodies conjugated with Alexa 488 or Alexa 550 were incubated for 90 min after primary antibodies, and sections were then washed with PBS and covered with Prolong-gold anti-fade mounting solution with nucleotide fluorescent dye 4',6-diamidino-2-phenylindole (DAPI; Invitrogen, Grand Island, NY). The fluorescent staining was photographed under fluorescent microscopy for cellular analysis (Leitz Orthoplan2 microscope; Ernst Leitz GMBH, Wetzlar, Germany) or confocal microscope (Olympus, Center valley, PA) for intra-nucleus analysis.

### Densitometry Analysis

For density analysis, pictures were taken using a Leitz Orthoplan 2 microscope with a Spot RT color camera (Diagnostic Instruments, Inc., Sterling Heights, MI). Bright-field images were taken with consistent setup and exposure time for each antibody staining. Immunostained images were converted to the 16-bit color format, and staining intensity was measured using Image J (National Institutes of Health, Bethesda, MD). Calibration was set based on 256 levels of the gray scale. Regions of interest (ROI) were picked by outlining the morphological areas at same level of brain section, e.g., whole hippocampus or dentate gyrus. For the measurement of subregions of hippocampus, a boxed region of equal dimensions from each cell layer was selected, and staining optical densities (OD) of the cells in ROI were compared among Chow, PF and Alc groups. Five brains from different dams were used from each treatment group and age. Statistical analyses were performed with one-way ANOVA followed by t-test using Prism software Version 4 (GraphPad Software Inc., San Diego, CA). All data were presented as Mean± SEM.

## Results

### 1. DNA methylation program in developing hippocampus

**Figure 1:**
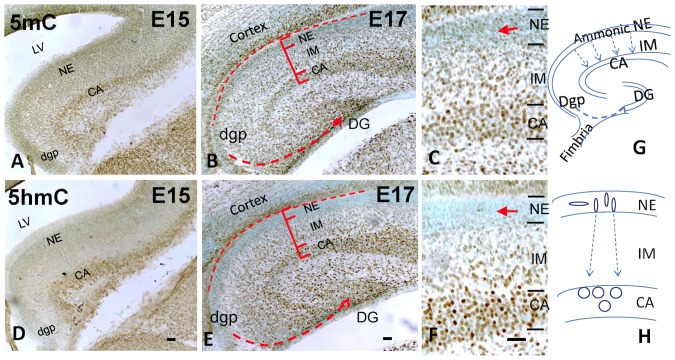
DNA methylation program of developing hippocampus during its early formation from E15 to E17. Cartoon (G,H) shows the differentiation processes where neuroepithelial (NE) cells migrate into intermediate zone (IM) and arrive in Cornus Ammonis (CA) to become pyramidal neurons; while dentate gyrus neuroepithelium (dgp) migrate through a long journey from lateral hippocampal primodium towards dentate gyrus (DG) and become granule cells. The DNA methylation formed an integral program in these differentiating cells. First progenitor cells all acquire DNA methylation to begin their differentiation. The immunostaining (brown DAB coloration) shows that the 5mC appears ahead of 5hmC as shown in E15 (A,D) and E17 NE (B,E) layer (enlargement, C,F, arrow). There is temporal increment of both methylation marks in CA (pyramidal layer) and developing dentate gyrus (DG) from E15 (A, D) to E17 (B,F). There is also a spatial increment of both 5mC-im and 5hmC-im from NE to IM and to CA at E17 (B,E; higher magnification in C,F, respectively). Moreover, the 5mC-im and 5hmC-im increase as the migration of granule cells from dentate neuroepithelium to the dentate primordial (B,E, dotted line). LV: lateral ventricle. Scale bar: A,B,D,E = 100 µm; C,F = 100 µm.

**Figure 2:**
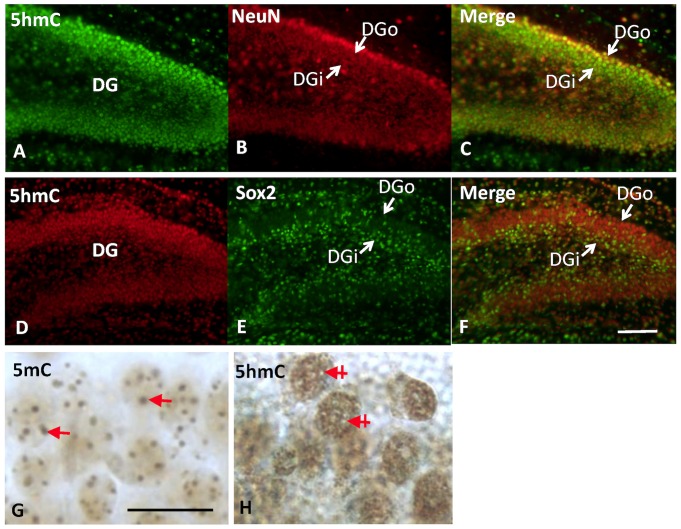
The close association of 5hmC mark with *Outside-In* pattern of neuronal differentiation in dentate gyrus. The immunofluorescent double staining reveals that the 5hmC (green in A and C) appeared in maturing or matured neuron as indicated by distribution and co-localization with NeuN (red, B and C), but not in neural progenitor cells marked with Sox2 (green in E and F vs 5hmC red in D and F) in P7 dentate gyrus. When observed through time course, the initiation of neural differentiation is synchronized with the appearance of 5hmC and is demonstrated by co-localization of 5hmC-im with NeuN^+^ cell in DGo (more mature than DGi), while not with Sox2+ cells in DGi. There is also chromatic translocation of the DNA methylation marks during differentiation, in which the 5mC and 5hmC separate in the nucleus of dentate granule cells (bright field). The 5mC is packed in large granule (G, arrow) and colocalized with DAPI dense area (not shown) in chromatin, while 5hmC is distributed in DAPI-light area complementary to the 5mC^+^ area (H, cross-arrow indicates 5mC area). DGo: dentate gyrus outer layer; DGi: dentate gyrus inner layer. Scale bar: A–F = 100 µm; G, H = 50 µm.

A patterned epigenetic progression was evident in the developing hippocampal Cornu Ammonis (CA) 1 through CA4. A temporal increment of 5mC and 5hmC-immunostaining (im) was observed from E15 to E17 in hippocampal pyramidal layer, as well as neuroepithelial layer (see **Figure 1A,D**). Meanwhile, there was also a spatial, cell-stage dependent gradation of DNA methylation in the developing hippocampus. At E17, the neural stem cells in the ammonic neuroepithelium (NE) layer were composed of spindle-shaped self-renewal neural stem cells as indicated by the expression of neural stem cell marker Sox2 and proliferation marker Ki67 (**Figure 1H**). These NE cells were either devoid or had only low levels of 5mC or 5hmC. As the NE cells committed to neural fate progression and begun differentiation, they migrated out radially, presumably along glial processes through the intermediate zone, and finally settled in the stratum pyramidale. These destined cells express neuronal marker NeuN and settled in an *inside-out fashion* (the early arrival differentiated cells settled in the inner layer, and the late arrival cells settled on the top of the earlier-arrived cells) (**Figure 1G,H**). Noticeably, the 5hmC-im was marked in the cells hours to a day behind the 5mC-im mark appeared (**Figure 1C,F**). It was the appearance of 5hmC which marked the cells undergoing migration. These migrating cells increased 5hmC as well as 5mC throughout their journey towards stratum pyramidale (**Figure 1B,C,E,F**). After settling in the CA primodium, these resident pyramidal neurons further matured by reducing 5mC methylation, and meanwhile went through a translocation of 5mC and 5hmC within the nucleus (see next stage). At P7, the maturing pyramidal neurons were NeuN+ and expressed both 5mC and 5hmC. However, there was a characteristic chromatic separation of the 5mC and 5hmC in the nucleus. The 5mC mark was preferentially clustered in the heterochromatic regions, which localized in the DAPI dense area, whereas 5hmC was more scattered in the euchromatic regions coinciding with the DAPI-sparse area (**Figure 2G,H**). Region wise, there was a general progression of maturation from CA1 towards CA4. There was also a progression of the above described 5mC and 5hmC in the same order.

Epigenetics also takes an orderly progression through the formation of dentate gyrus (DG). At E17, the *Primary Dentate NEs* which were located lateral to the ammonic CA in the hippocampal primodium (see **Figure 1G**), just began to migrate medially to the *Secondary Dentate Matrix* (an intermediate regions within hippocampal primodium), and then further migrated to the target dentate regions forming a small granule cell cluster of angular dentate primodium (**Figure 1G**, dotted arrow). These NE exoduses first lost Sox2-im and Ki67-im by acquiring 5mC followed with 5hmC on the journey, and became granule cells expressing NeuN at the destined area. At P7, an *outside-in settling* was formed as these exodus cells reached the target dentate layer––the earliest arrivals, the most matured granule cells, formed the outside shell of the granular layer, and became NeuN^+^ (**Figure 2A-C**). The late-arriving cells settled beneath them, expressing neuroblast marker doublecortin (Dcx).

**Figure 3:**
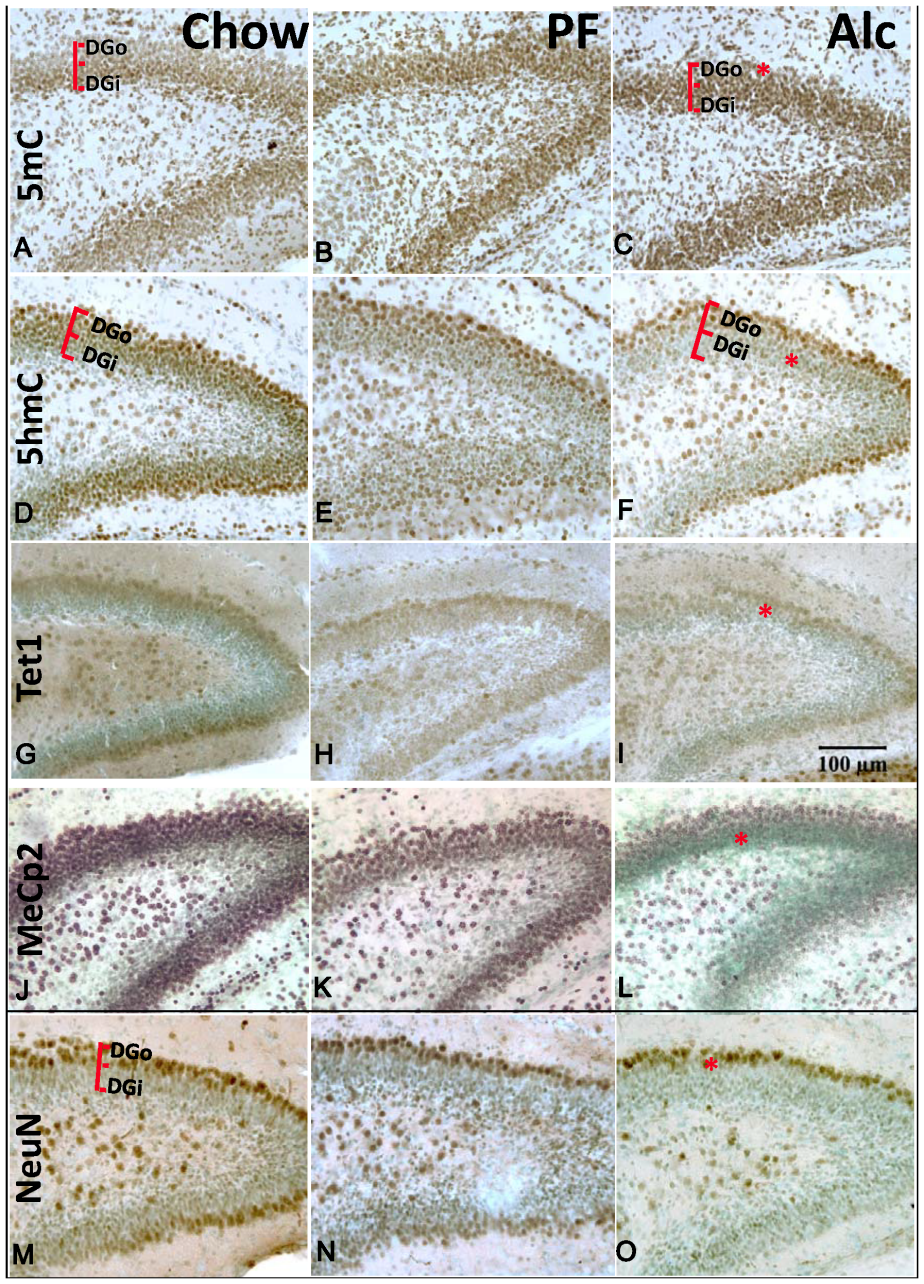
DNA methylation marks and neuronal maturation in P7 dentate gyrus (DG) and effect of alcohol (see also quantitation in Figure 8). Immunostaining of DNA methylation marks, 5mC (A–C) and 5hmC (D–F), 5hmC-converting enzyme TET1 (G–I), DNA methylation binding protein MeCp2 (J–L), and mature neuronal marker NeuN (M–O). There is a methylation gradation correlating with the *Outside-In* pattern of neuronal maturation in DG (M), in which the 5mC-im decreases as granule cells mature (A: DGi>DGo), while 5hmC-im increases as granule cells mature (D: DGi<DGo). Alcohol exposure derailed the reduction of 5mC in DGo (C, star) and the acquisition of 5hmC in both DGi and DGo (F, star), which is accompanied by the delayed maturation of DG as indicated by decreasing the NeuN+ cells (O, star). There is no significant difference between PF and Chow. The TET1 protein expression is closely associated with the expression of 5hmC (DGi<DGo), which was also reduced by Alc (I, see star). The expression of MeCP2 known to correlate with neuronal maturation was also reduced by alcohol (L, star). DGo: dentate gyrus outer layer; DGi: dentate gyrus inner layer. Scale bar: all = 100 µm.

Meanwhile, as the DG primodium continued its formation, a new source of neural progenitor cells expressing Sox2 and Ki67 was established at the most inner layer (**Figure 2E**), *the subgranular layer (SGZ)*, which was derived from previous *Secondary Dentate Matrix.* Very similar to CA, these new dentate progenitor cells launched their neural fate by acquiring first the 5mC, while 5hmC was absent or low. The 5hmC appeared when progenitor cells set off for migration outward (some inward-migrating cells became interneurons). This new wave of migrating cells increased 5hmC-im while decreased 5mC through the journey towards the outer dentate shell in both upper and lower limbs of DG. At this stage, a clear reverse gradation of 5mC and 5hmC marks was formed in the DG, well correlated with the maturation of granule cells (**Figure 3A, D, M**). A similar translocation of 5mC and 5hmC in association with chromatin also occurred during the differentiation process. In the nucleus of horizontal or spindle shaped progenitor cells, the 5mC was diffused, while in the mature sphere-shape cells in the target layer, the 5mC mark became condensed and granulated in the nucleus. The double fluorescent staining showed co-localization of 5hmC with NeuN^+^ cells, but not Sox2^+^ cells in dentate gyrus (**Figure 2A-F**). TET1 expression was closely associated with 5hmC-im, but not 5mC-im in DG, in which TET1-im was higher in the outer layer than in the inner layer (**Figure 3G**). The DNA methylation binding protein MeCp2 showed similar expression gradation as seen for NeuN and TET1- high in the outer granule layer and low in the inner layer (**Figure 3J**).

The progenitor cells in the subgranular layer lasted throughout adulthood; the progression of the neural differentiation of these progenitor cells unfailingly went through the DNA methylation program described above regardless of age.

### 2. Effect of the alcohol exposure

**Figure 4:**
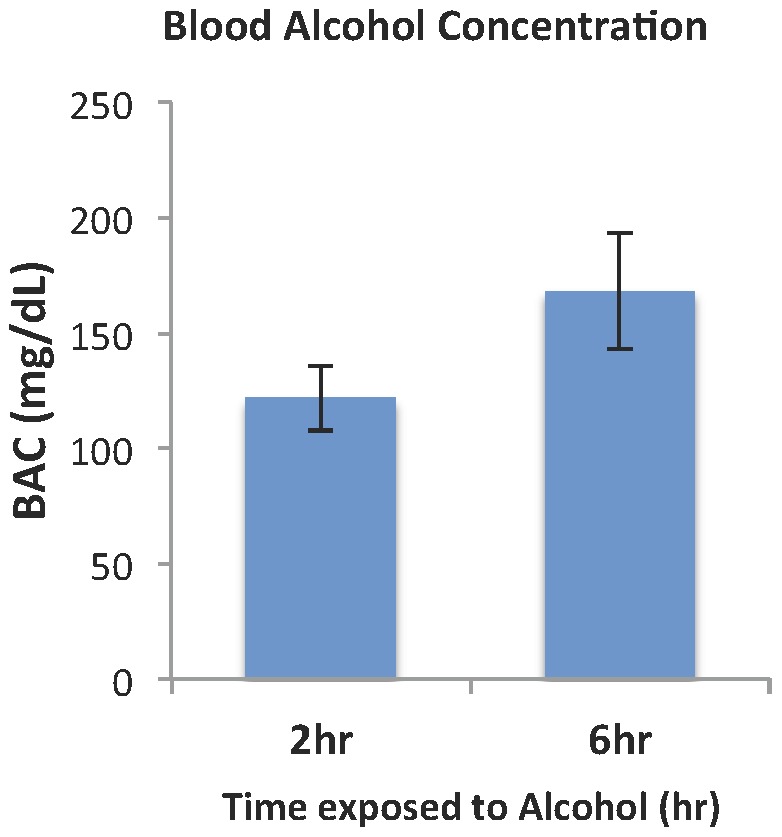
Blood alcohol concentration (BAC) 2hrs and 6hrs after alcohol diet supply at beginning of the dark cycle. The BAC was collected from a group of females independent from the group of epigenetic and phenotypic studies (N = 6). Tail blood was collected for measurement of BAC every other day for a total of 3 collections for each animal. All data were presented as Mean ± SEM.

**Figure 5:**
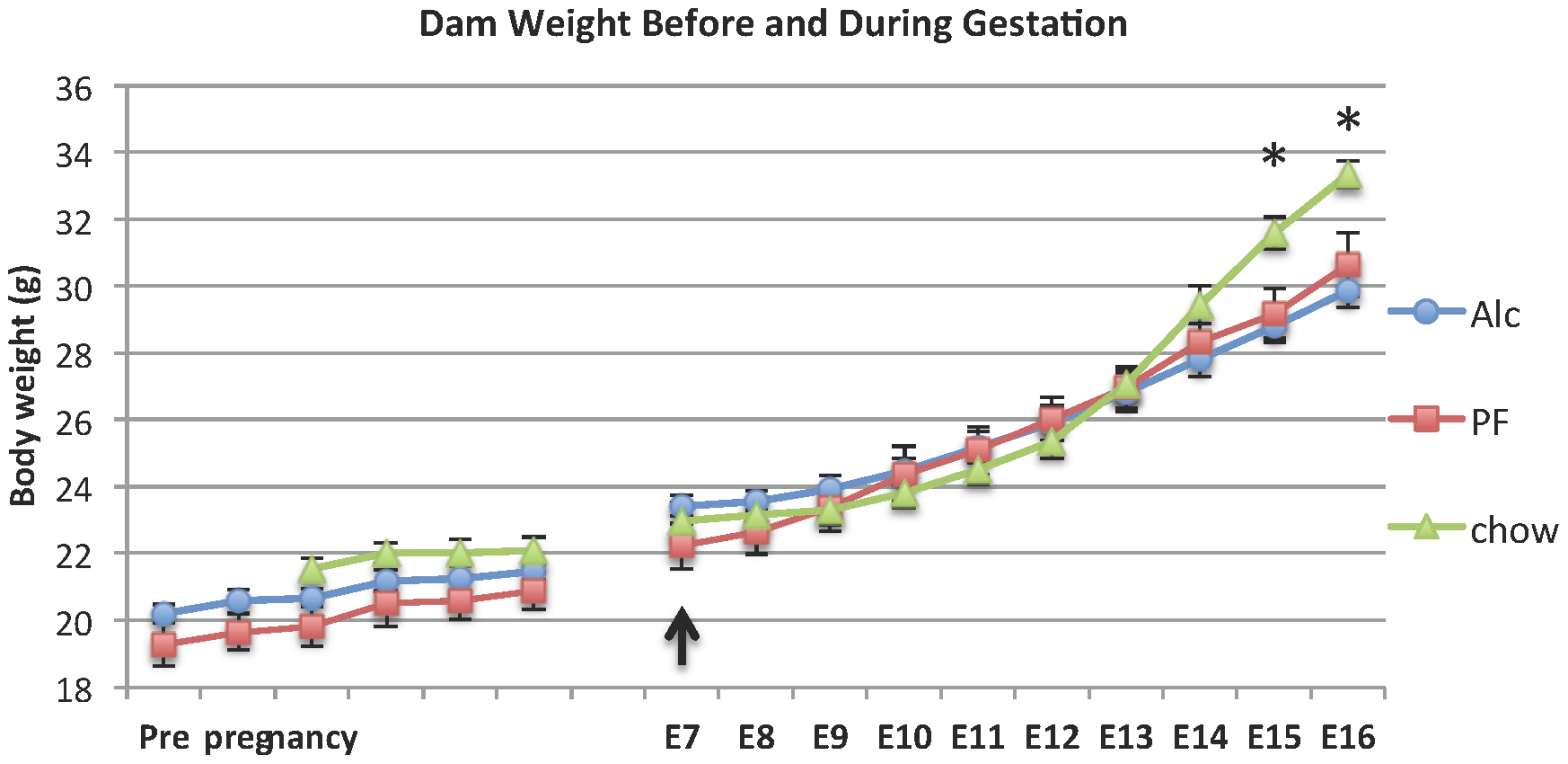
Dam weight before mating and throughout gestation 7-16 days of Chow, PF and Alc groups. Before mating, the Alc and PF groups were pre-treated with liquid diet for 7 days, while Chow group were on chow pallets diet throughout the time course. The treatment for 4%(v/v) alcohol or pair-fed liquid diet was administered from embryonic day (E) 7 to E16. Arrow: day treatments started. N =  Chow (5), PF (5), Alc (7). *One-way ANOVA: P<0.05. All data were presented as Mean ± SEM.

The blood alcohol concentration (BAC) in the current treatment paradigm peaked at ∼120–160 mg/dL (**Figure 4**) is similar to that of our previous study [31]. Under the treatment, there was no significant difference in dam body weight from the start of treatment (E7) to E14 among Chow, PF and Alc groups (One-way ANOVA, P>0.05) (**Figure 5**). However, dam body weight of the PF and Alc groups were lower than the Chow group at E15 and E16 (One-way ANOVA, F[2], [13]<0.005), while there was no difference between PF and Alc groups (t-test, P>0.05) (**Figure 5**).

**Figure 6:**
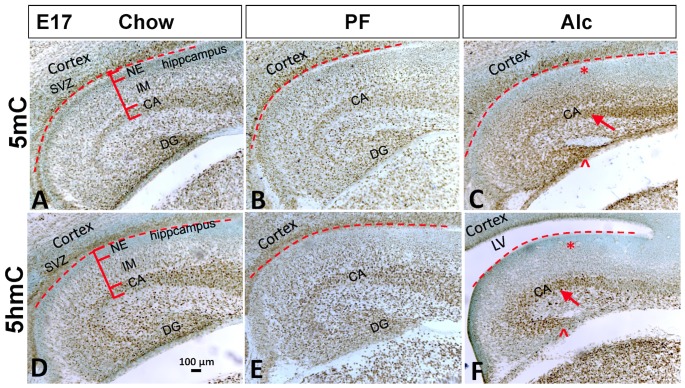
Alcohol altered DNA methylation while retarded the hippocampal formation at E17. Alcohol exposure reduced hippocampus size (C,F) as compared to that of Chow (A,D) and PF (B,E) (see quantitation in Table 1), increased lateral ventricle space, and diminished dentate gyrus size (C,F, arrowhead; also see Table 1). Alcohol exposure expanded NE thickness (see quantitation in Table 1), reduced both 5mC-im and 5hmC-im in NE (C, F, stars), while increased both 5mC-im and 5hmC-im in CA (C,F, arrow) (see quantitation in Table 2). There was no significant different between PF and Chow. Dotted line: separation of SVZ of cortex from NE of hippocampus. LV: lateral ventricle; SVZ: subventricular zone. NE: neuroepithelium; IM: intermedium zone; CA: Conus Ammonis; DG: dentate gyrus. Scale bar: all = 100 µm.

**Figure 7:**
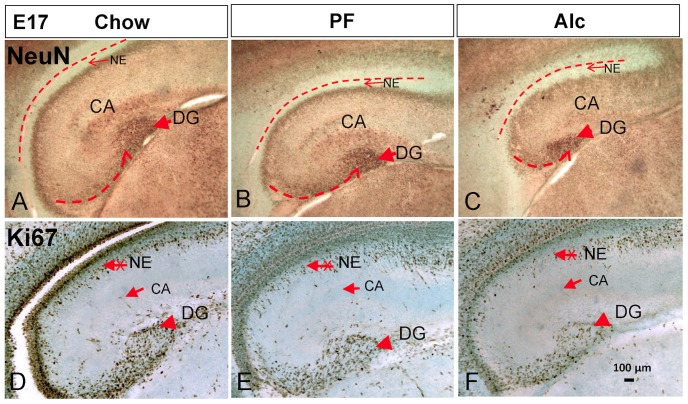
Alcohol reduced the proliferation and maturation of hippocampal cells at E17 (Also see quantitation in **Table 1d,e** **).** Alcohol reduced NeuN^+^ cell number in NE, CA and DG (C, arrowhead) as compared to that of Chow (A) and PF (B), as well as reduced dentate granule cell migration distance (comparing A,B,C, dotted line). The proliferation marker Ki67-im was reduced in Alc groups in NE (F, crossed arrow) compared to Chow (D) and PF (E), and DG (comparing D,E,F, short arrows). Scale bar: all = 100 µm.

Alcohol delayed the maturation of hippocampus by approximately 1 day, as indicated by comparison between E17 Alc to E16 Chow embryos. The hippocampus size was reduced significantly in the Alc group as compared to Chow and PF groups (**Table 1a**), and the lateral ventricle was expanded in the Alc group (**Figure 6F**). The thickness of both the (undifferentiated) ammonic NE and the primary dentate NE layers was increased in alcohol group as compared to those of Chow and PF groups (**Table 1c**), with a concomitant fall in the 5mC and 5hmC levels (**Table 2a**, **Figure 6C**). The rate of proliferation was reduced by alcohol, as indicated by less Ki67^+^ cells (**Table 1d**). Outside the NE layer, there was a slight reduction in the ammonic cell migration, as well as reduced numbers of migrating cells in the intermediate zone (**Figure 7A**–**C**). The 5mC-im intensity was higher in the cells of the intermediate zone (not timely reduced) while the 5hmC-im intensity was lower (not timely increased) in Alc as compared with PF and Chow groups (**Table 2b**). The thickness of formed stratum pyramidale was thinner in alcohol groups as compared with PF and Chow groups; and both the 5mC-im and 5hmC-im in the CA1 region were significantly higher in Alc group, which were normally reduced as neuron became matured (**Table 1c** **,** **Figure 6C,F**, arrow).

**Table 1:** Phenotypic measurements of E17 hippocampus in Chow, PF and Alc groups.

E17	Chow	PF	Alc
a. Hippocampus size (10^3^ µm)	392.3±16.0	387.4±22.0	280.0±20.0 ** ^#^
b. DG size (10^3^ µm)	24.3±1.0	23.9±2.1	15.7±1.1 ** ^#^
c. NE thickness ( µm)	51.1±3.8	50.7±2.9	68.7±4.9* ^#^
d. Ki67-im (in NE)	143.6±7.4	152.4±3.5	109.5±13.7* ^#^
e. NeuN-im (in DG)	178.9±1.4	169.2±6.6	152.1±5.1* ^#^

* P<0.05; **P<0.005 compare Alc to Chow; ^#^P<0.05 compare Alc to PF. ?P<0.05 compare PF to Chow. N = 4. Data presented as Mean±SEM. NE: neuroepithelial layer; DG: dentate gyrus.

**Table 2:** DNA methylation immunostaining (im) intensity in E17 hippocampus.

	a. NE			b. IM			c. CA1		
E17	Chow	PF	Alc	Chow	PF	Alc	Chow	PF	Alc
5mC-im	168.1±5.3	152.1±3.2?	150.9±6.9*	139.9±5.7	132.3±9.8	159.0±5.3* ^#^	157.8±6.4	151.8±11.7	173.7±1.8*
5hmC-im	116.9±6.4	127.2±3.6	84.4±5.4* ^#^	113.52±5.7	134±5.2?	112.1±4.8^#^	144.7±2.6	163.6±3.6?	167.1±3.0**

* P<0.05, **P<0.005 (Alc vs Chow); ^#^P<0.05 (Alc vs PF); ?P<0.05 (PF vs Chow). N = 4 each. Data presented as Mean±SEM. NE:neuroepithelial layer; IM: intermediate zone.

In the developing DG of Alc embryos, the size of dentate gyrus was significantly reduced (**Table 1b**), the migration distance was shortened and the secondary and tertiary matrix was diminished (**Figure 7A**–**C**, dotted line). The number of NeuN^+^ granule cells that reached primordial DG was also reduced (**Figure 7A**–**C**, arrowhead) along with lower level of NeuN-im (**Table 1**e). The 5mC-im intensity of differentiated granule cells that arrived in dentate primodium remained high (un-tapered) in Alc as compared with that of PF and Chow groups (**Figure 6C**, arrowhead). The effect of alcohol on TET1 protein was similar to that of 5hmC. The TET1-im was reduced in NE as well as in intermediate zone, while slightly increased in stratum pyramidale.

The differential DNA methylation upon alcohol treatment progressed in the CA until they all reached their plateau by P7. However, at P7 the DG was continuously affected by the prenatal alcohol exposure (**Figure 3** **and** **8**). The total number of NeuN-expressing cells was significantly reduced by 45.6% (t-test, P<0.001) in the Alc group compared to that of Chow group; no difference when comparing Chow to PF groups (one-way ANOVA: F(2,18) = 35.8452, P<0.001) (**Figure 3M**–**O**). In order to more closely analyze the methylation level in differentiating cells within the DG, the intensity of 5mC-im and 5hmC-im was measured separately in the inner granule layer (DGi) and the outer (shell of) granule layer (DGo) (**Figure 3A**–**F**). The 5mC-im was significantly higher (not entering the programmed reduction) in DGo (One-way ANOVA: F(2,6) = 5.26, P<0.05) in the Alc group as compared with that of PF and Chow, while no difference was observed in DGi among treatments (One-way ANOVA, F(2,9) = 1.037, P>0.05). The 5hmC-im was reduced significantly by alcohol in both DGo and DGi (One-way ANOVA: F(2,6) = 5.26, P<0.05). Post-hoc analysis showed no difference between PF and Chow, however, the Alc group was significantly different from the other two (t-test, P<0.005).

**Figure 8:**
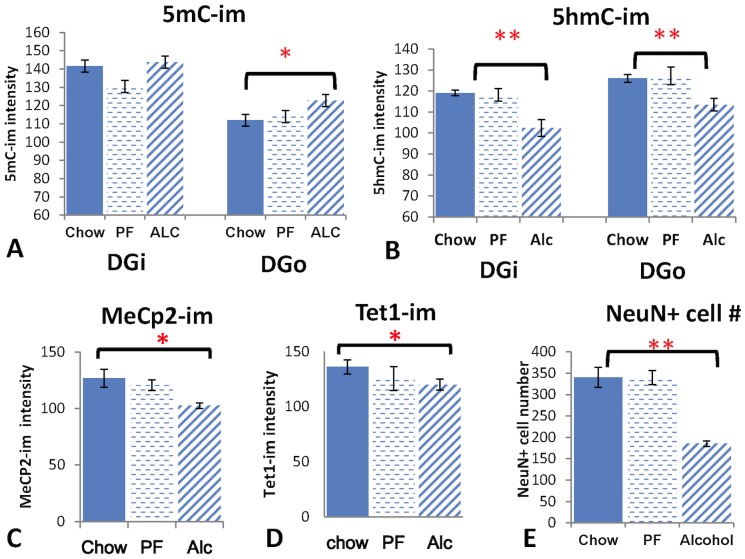
Statistical analysis of the immuno-intensity of neuronal marker NeuN and methylation marks (5mC, 5hmC, TET1 and MeCp2) in P7 DG in Chow, PF and Alc groups. N = 5, data presented as Mean±SEM. *P<0.05; **P<0.005.

Along with the alteration of 5mC and 5hmC, the TET1-im and MeCp2-im in DG were also altered upon alcohol treatment (**Figure 3**). The MeCp2-im was significantly reduced in alcohol treatment (**Figure 3J-L**, one-way ANOVA: F(2,12) = 8.907, P<0.005) followed by a Bonferroni post-hoc analysis determined that all three groups were significantly different from one another. TET1-im was also reduced in Alc group as compared to PF controls (t-test, P<0.02) (**Figure 8**).

## Discussion

### Principle of DNA Methylation Program during hippocampal development

Compared to the DNA methylation program (DMP) in neural tube development [8], the DMP in maturing hippocampus is more intricate. However, the principle upholds. The hippocampal DMP with 5mC, 5hmC and DNA methylation binding proteins step-by-step along neuronal differentiation and maturation is deliberated below. There are three independent lines of neuronal lineage within hippocampal formation, each with an independent DMP.

**Figure 9:**
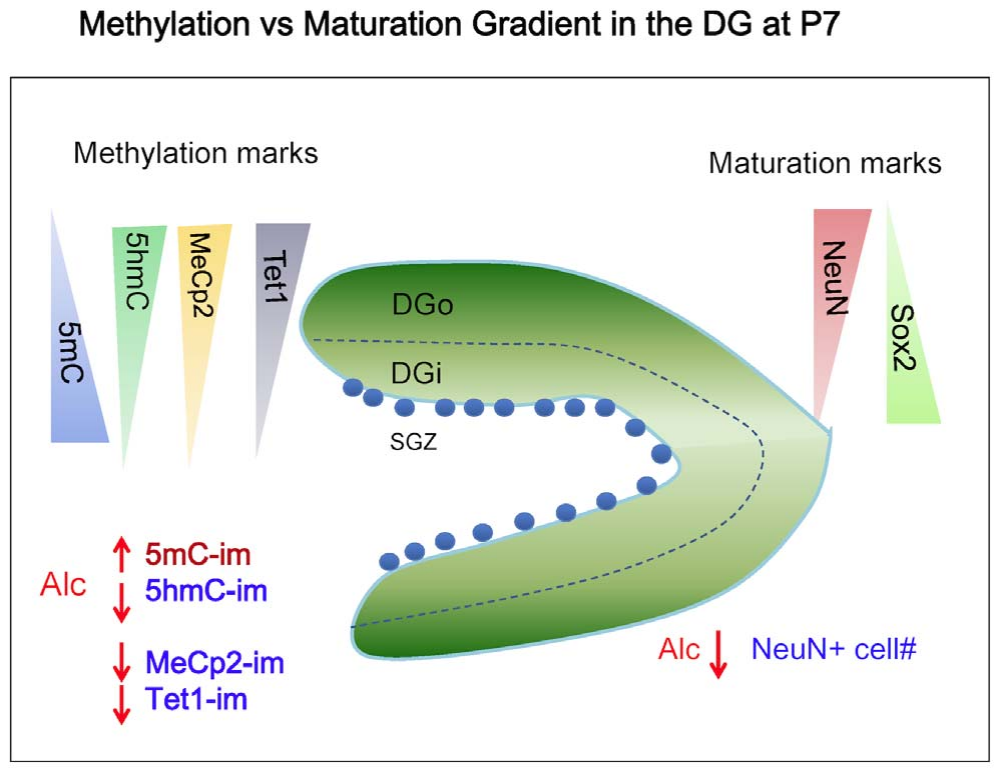
Illustrated summary of the correlation of DNA methylation with neuronal maturation in P7 dentate gyrus and the effect by alcohol. In the developing DG, migrating granule cells arrive at their target regions in an *Outside-In* pattern. The outermost layer (DGo) is composed of most mature neurons (NeuN^+^), while the inner layer (DGi) is composed of younger neural progenitor cells (NeuN^-^). A subgranular layer (SGZ) starts to form composing of neural progenitor cells (Sox2^+^) at the innermost of each dentate limb. There is a correlating methylation gradation along with maturation of granule cells that the 5hmC-im increases while 5mC-im reduces towards the outer dentate shell of DG. The expression of TET1 and MeCp2 were closely associated with the DNA methylation gradation. Alcohol exposure disrupts the DNA methylation machinery (i.e. 5mC, 5hmC, MeCP2, and TET1) and delays the maturation of DG (e.g. NeuN).

First, during the CA formation, similar to neural tube development, the neural progenitor cells in the ventricular zone possess extremely low methylation marks until they ceased proliferation. Prior to embarking differentiation from a renewal state, the differentiating ammonic NE acquired or drastically increase 5mC. The 5hmC sets in when migration begins in NE and in intermediated zone which continues its migration into intermediate zone. After settling in the pyramidal layer in an orderly inside-out pattern, the pyramidal neurons reduce 5mC while continuing to increase 5hmC in the order of their maturity within the CA layer in an “inside-out pattern”. Second, in the primary DG development (progenitor cells from primary dentate matrix), an identical DMP occurred along the path of granule cell differentiation in its own time course. Thus, the progression of the DMP is not synchronized to the age of the brain, but to the stage of differential state of each lineage of brain cells. This idea continues and is supported by the third lineage of progenitor differentiation in the subgranular layer of DG where a new pool of progenitor differentiation remains throughout the life. Not only is DMP progressed temporally, but also spatially as evidenced in the NE layer to the final destination in the target layer. This is particularly supported even when the arrangement of the granule cells within the granular layer is, on the contrary, reversed in an “outside-in” pattern [32], [33]. The DMP progressed in a precise “outside-in” pattern slightly ahead of (or leading) the progression of maturation within the layers (**Figure 9**). Thus, three lineages of neuronal development occurred independently in their own temporal pace and spatial location followed the same DMP invariably within the hippocampal formation.

The functional aspect of the DMP is hinted by the coordinated translocation of the 5mC and 5hmC in association with the euchromatin and heterochromatin along the passage of the DMP. The 5mC started to appear as a fine granular DNA cluster in the nucleus, likely to silence the group of genes that maintain stem cell properties. The 5mC is translocated on the chromatin from sparsely distributed forms within nucleus in early differentiating cells to condensed granulated forms associated with heterochromatin, while 5hmC to euchromatin is highlighted in the mature neurons where neuronal specialization is settled and maintained. Increasing evidence indicates that 5hmC is sliding toward transcriptional activation or at least at bivalent states [19], [22], [34]. Besides the chromatic relocation, one more finding worthy of mentioning is that the maturing neuron gains the 5hmC marker, while reduces the 5mC marker, as shown from the migrating granule cells toward the settled granule layers. This is consistent with other reports that 5hmC accumulates in neurons during development and aging [21], [35] and is supportive towards the role of 5hmC in neuronal maturation.

### Alcohol on DNA Methylation Program

Alcohol exposure, through altering methyl donor metabolism, has been shown to affect global [29], [30] and gene specific methylation [36], [37]. The 4% (v/v) alcohol we adapted reflected a moderate high chronic drinking paradigm in humans, with BAC reached 120–160 mg/dL. Similar level of BAC during gestation stages in rat has been shown to induce spatial learning deficits and despair behaviors [2], [38], [39]. In this study, we further demonstrated that chronic moderate alcohol exposure from the late 1^st^ trimester throughout the 2^nd^ trimester led to significant alteration in DMP progress (**Figure 9**) in the three developmental lines described above.

First, alcohol delays the acquisition of 5mC as well as 5hmC in neural progenitor cells in the NE. The embarking of DNA methylation (5mC and 5hmC) at the start of neural fate determination is likely associated with the silencing of stem cell maintaining genes, (e.g. *POU5F1, Ddah2* reported being turned off during neuronal differentiation*),* and the expression of neural differentiation genes (e.g. *Jag1* and *Tcf4* reported increased during neuronal differentiation*)* [40]. Alcohol preventing the onset of gene methylation has been shown to retard the neural differentiation [11] or deviate towards glial properties [41].

Another significant alteration is that in early maturing neurons (both stratum pyramidale and dentate granule layer), the regression and intra-nucleus relocation of 5mC, as well as accumulation of 5hmC are hindered by alcohol exposure. Considering the diversification of 5mC and 5hmC during maturation in gene promoters and gene bodies [42], the functional results of alcohol exposure could be a disruption of recruitment of chromatin modifying enzymes, e.g. DNMT3, Sirt1, G9a [43] that alter epigenetic marks, and further an alteration in transcription. The altered DNA methylation marks might also affect their binding protein to recruit transcription factors and other histone codes. We have observed a reduced expression of MeCp2 in these early maturing neurons.

Furthermore, we demonstrated a lasting effect of prenatal alcohol exposure on DNA methylation through newborn age (postnatal day 7 in mice). After alcohol exposure has been removed for the whole 3^rd^ trimester period, we still observed significant DMP alteration in developing dentate gyrus. It might be explained by the biochemical inhibition of methyl-donor reactions, and/or the lack of 5hmC-converting enzymes (TET1) from the previously exposed alcohol. These epigenetic discrepancies appear to line up after neurons are matured (e.g. pyramidal neurons at P21), where differentiation processes is completed. These results indicate that the alcohol affected DNA methylation during active differentiation and its influence on methylation machinery are likely consequential to the developmental process of the hippocampal neurons as noted by many reports [21], [44], [45], [46].

### Alcohol and epigenetic prospect of developmental teratology

Although alcohol affected the methylation program that correlated with a developmental delay in hippocampus in vivo, whether the aberrant DNA methylation program leads to the developmental delay remains to be seen. However, studies showed that ablation or overexpression of methylation machinery, e.g. DNMTs or TDG proteins, was embryonically lethal [47], [48], [49]. Mutations in methyl-binding proteins and imprinting genes are related to the onset of developmental deficits such as Rett syndrome, Angleman syndrome and Prader-Willi syndrome [26], [50], [51]. Our previous studies showed that 5-azacytidine a DNMT inhibitor for DNA methylation, retarded the embryonic growth during early neurulation [8]. Similarly, choline deficiency (lack of methyl donor) reduced global DNA methylation, and gene-specific methylation at the neuroepithelium in mice hippocampus, and impairs memory performance [52]. On the other hand, supplementation of choline can improve the developmental deficits in hippocampus neural systems [53], [54], [55]. Thus, it is plausible that deregulation of DNA methylation mediates the alcohol teratology in FASD model. Ongoing studies are investigating the DNA methylation changes and transcription at the gene level. Nevertheless, this study provides the first demonstration that the DNA methylation program is an upstream orchestrated order during hippocampal differentiation and maturation, and alcohol disrupts the intricate order while retards the normal development.
